# Dexmedetomidine Protects Mouse Brain from Ischemia-Reperfusion Injury via Inhibiting Neuronal Autophagy through Up-Regulating HIF-1α

**DOI:** 10.3389/fncel.2017.00197

**Published:** 2017-07-06

**Authors:** Cong Luo, Ming-Wen Ouyang, Ying-Ying Fang, Shu-Ji Li, Quan Zhou, Jun Fan, Zai-Sheng Qin, Tao Tao

**Affiliations:** ^1^Department of Anesthesiology, Nanfang Hospital, Southern Medical UniversityGuangzhou, China; ^2^Department of Anesthesiology, The Fifth Affiliated Hospital, Southern Medical UniversityGuangzhou, China; ^3^Department of Neurobiology, School of Basic Medical Sciences, Southern Medical UniversityGuangzhou, China

**Keywords:** dexmedetomidine, cerebral ischemia, neuroprotection, autophagy, HIF-1α

## Abstract

Stroke is the leading cause of death in China and produces a heavy socio-economic burden in the past decades. Previous studies have shown that dexmedetomidine (DEX) is neuroprotective after cerebral ischemia. However, the role of autophagy during DEX-mediated neuroprotection after cerebral ischemia is still unknown. In this study, we found that post-conditioning with DEX and DEX+3-methyladenine (3-MA) (autophagy inhibitor) reduced brain infarct size and improved neurological deficits compared with DEX+RAPA (autophagy inducer) 24 h after transient middle cerebral artery artery occlusion (tMCAO) model in mice. DEX inhibited the neuronal autophagy in the peri-ischemic brain, and increased viability and decreased apoptosis of primary cultured neurons in oxygen-glucose deprivation (OGD) model. DEX induced expression of Bcl-1 and p62, while reduced the expression of microtubule-associated protein 1 light chain 3 (LC3) and Beclin 1 in primary cultured neurons through inhibition of apoptosis and autophagy. Meanwhile, DEX promoted the expression of hypoxia-inducible factor-1α (HIF-1α) both *in vivo* and *in vitro*, and 2-Methoxyestradiol (2ME2), an inhibitor of HIF-1α, could reverse DEX-induced autophagic inhibition. In conclusion, our study suggests that post-conditioning with DEX at the beginning of reperfusion protects mouse brain from ischemia-reperfusion injury via inhibition of neuronal autophagy by upregulation of HIF-1α, which provides a potential therapeutic treatment for acute ischemic injury.

## Introduction

Dexmedetomidine (DEX), a highly selective α_2_-adrenoceptor agonist, is primarily applied in post-anesthesia care unit and neurological intensive care unit for sedation and analgesia (Li et al., 2016). Increasing evidences have shown that DEX is protective after ischemia-reperfusion injury of several vital organs (Cai et al., 2014), including ischemic cerebral injury. Early adaptive response to ischemic insults depends on the change of gene/protein expression within the injured areas to regulate the opening of chemical channels, neurotransmitter release to minimize brain damage (Acker, 2004). It has been found that the activation of phosphatidylinositol 3-kinase (PI3K)-Akt pathways via the α_2_-adrenoceptor are involved in DEX-induced protection against renal ischemia/reperfusion (Gu et al., 2011; Zhang et al., 2012). DEX has been shown to defend against ischemia-induced brain injury through activation of extracellular signal-regulated protein kinase 1 and 2 (ERK1/2) pathways or PI3K-AKT pathways via α_2_-adrenoceptor-independent mechanisms (Dahmani et al., 2008). Some of DEX-induced neuroprotective functions are mediated via the α_2_-adrenoceptor subtypes (Ma et al., 2004). In addition, some studies have reported that DEX preconditioning effectively reduces the inflammatory response of myocardial, renal and spinal cord ischemia by downregulation of high-mobility group protein B1 (HMGB1)/toll-like receptor 4 (TLR4) pathways (Gu et al., 2011; Rong et al., 2017; Yang et al., 2017). Meanwhile, DEX has been reported being protective in brain, intestinal, renal and lung by inhibiting apoptosis, necrosis (Engelhard et al., 2003; Gencer et al., 2014; Cui et al., 2015; Sun et al., 2015) and inhibition of autophagy in lung and kidney ischemic reperfusion injury (Lempiäinen et al., 2014; Xie et al., 2015). However, whether DEX regulates autophagy in cerebral ischemia-reperfusion injury is still unknown.

Autophagy is a cellular physiological and pathological process for degradation of injured intracellular organelles, proteins and other cell components to maintain homeostasis. Autophagy is activated after focal cerebral ischemia in mice brain tissue (Degterev et al., 2005), cortical neurons and astrocytes (Xu and Zhang, 2011). Application of an *in vivo* imaging technique, Tian et al. (2010) delineated the autophagy at different time points after transient middle cerebral artery (MCA) occlusion (tMCAO), with a peak of autophagy at 1 day after tMCAO. In terms of area and neural cell type, autophagy predominantly exists in the peri-ischemic areas and in neurons. Consistently, higher level of autophagy is found in neurons than astrocytes in a neonatal cerebral ischemia model (Puyal et al., 2009). Notably, the role of autophagy in ischemic brain injury is being elucidated (Wei et al., 2012). Qin’s group found cortical neuronal injury was associated with activation of autophagy in the permanent focal cerebral ischemia and indicated that inhibition of autophagy may help reduce ischemic cerebral injury (Wen et al., 2008). However, their further research suggested that induction of autophagy through a brief focal ischemic preconditioning was neuroprotective after permanent cerebral ischemia (Sheng et al., 2010). In addition, preconditioning of 3-methyladenine (3-MA), an autophagy flux inhibitor, has been reported failed to reduce the neuronal cell death in cerebral ischemia (Wang et al., 2014). However, neuronal death is largely prevented by inhibition of autophagy in neonatal ischemic brain injury (Uchiyama et al., 2008). Therefore, whether autophagy is neuroprotective or pro-death in ischemic brain still needs further investigation.

In this study, we hypothesized that DEX may regulate neuronal autophagy in the cortex being neuroprotective after tMCAO *in vivo* and oxygen-glucose deprivation (OGD) *in vitro*. To test our hypothesis, we first observed the autophagy in neurons treated by DEX, or combined with autophagy inhibitor 3-MA or inducer RAPA both *in vivo* and *in vitro*. Our results showed that DEX was neuroprotective by inhibition of the cortical neuronal autophagy at the beginning of reperfusion. Furthermore, we found DEX-mediated inhibition of autophagy via up-regulating the expression of hypoxia-inducible factor-1α (HIF-1α).

## Materials and Methods

All experimental procedures were approved by the Southern Medical University Administrative Panel on Laboratory Animal Care, and experiments were conducted in accordance with the guidelines of Animal Use and Care of Southern Medical University. All efforts were made to minimize the number of animals stress and pain.

### Focal Cerebral Ischemia Model

Male C57/BL6 mice (8–10 weeks old, 22–25 g) were purchased from the Experimental Animals Center of Southern Medical University. tMCAO model was used as focal cerebral ischemia model (Liu et al., 2009). Briefly, mice were anesthetized with isoflurane (5% for induction and 1.5%–2.5% for maintenance). A 6-0 silicone rubber-coated nylon monofilament (YuShun Biological Technology Co. Ltd.) was inserted from a cut of the right external carotid artery, then monofilament was introduced into the internal carotid artery through the carotid bifurcation and advanced 9–11 mm to occlude the MCA for 90 min. Transcranial LDF (Moor VMS-LDF, Moor Instruments, United Kingdom) was used to measure the cerebral blood flow (CBF) in the MCA territory (1 mm posterior and 5 mm lateral to the bregma on the right parietal skull). A 75% or higher decrease in the regional CBF was considered as successful occlusion. Rectal temperature was maintained at 37 ± 0.5°C during entire surgical procedures by a heating pad. During the occlusion period, the anesthesia was disrupted and mice were placed back to their cages. Mice were re-anesthetized to remove the monofilament, ligate the cut and close the incision after 90 min occlusion. Mice in sham group adopted an exactly same procedure except the MCA occlusion.

### Drug Administration

DEX (Jiangsu Hengrui Medicine Co., Ltd., China; Gu et al., 2011), and rapamycin (RAPA, Sigma) were diluted or dissolved with 0.9% saline, then intraperitoneally injected into mice at dosages of DEX (25 μg/kg) and RAPA (80 μg/kg). 3-MA, Sigma-was dissolved in 0.9% saline and was injected intracerebroventricular into mice with 3 μl of a 20 mg/ml (Wang et al., 2014). At the onset of the reperfusion, the treatment groups, including tMCAO+DEX, tMCAO+3-MA, tMCAO+DEX+3-MA, tMCAO+RAPA and tMCAO+DEX+RAPA were administrated with DEX, 3-MA, DEX combined with 3-MA and RAPA and DEX combined with RAPA respectively. 2-Methoxyestradiol (2ME2, Selleck) was dissolved in 0.5% dimethylsulfoxide (DMSO) and intraperitoneally injected 30 min after tMCAO (Schaible et al., 2014). Sham and tMCAO groups without drug treatment were injected with the same volume of 0.9% saline or DMSO.

### Neurological Deficit Score

The neurological deficit scores of the mice were evaluated 1 day after tMCAO by researchers who were blinded to the experimental groups. The neurological grading scores range from 0 to 5 (0, no deficit; 1, forelimb flexion; 2, as for 1, plus decreased resistance to lateral push; 3, unidirectional circling; 4, longitudinal spinning or seizure activity; and 5, no movement; Jin et al., 2015).

### Infarct Size Measurements

Infarct size was measured 1 day after tMCAO. The mice were deeply anesthetized, decapitated and their brains (*n* = 8/group) were rapidly removed and sliced into six sections using a rodent brain matrix. The brain slices were stained with 2% 2,3,5-Triphenyltetrazolium chloride (TTC, Sigma) at 37°C for 15 min, then fixed in 4% paraformaldehyde for 1 day (Yang et al., 2013). The infarct size was analyzed using ImageJ (1.37v, Wayne Rasband, available through the National Institutes of Health) by researchers who were blinded to the experimental group. To exclude the effect of cerebral edema, the infarct size was normalized to the non-ischemic hemisphere and expressed as percentage of the contralateral hemisphere.

### Transmission Electron Microscopy

To examine the formation of autophagosomes and morphology changes in the neurons after tMCAO in cerebral cortex, transmission electron microscopy examination was performed as previously described (Liu et al., 2016). Briefly, mice were euthanized with an overdose isoflurane and transcardially perfused with icy PBS (pH 7.4) followed by 4% paraformaldehyde in PBS (pH 7.4) 1 day after tMCAO. Brain tissue (1 mm^3^) from the peri-ischemic area of cerebral cortex were fixed with ice-cold glutaraldehyde (2.5% in 0.1 M cacodylate buffer, pH 7.4) for 1 h, and post-fixed in OsO_4_ and embedded in epoxy resin. Ultrathin sections (70–80 nm) were stained with uranyl acetate and lead citrate and examined in a CM-120 electron microscopy (Philips, Holland).

### Tissue Preparation and Immunostaining

Twenty-four hours after ischemia onset, animals were sacrificed with an overdose of isoflurane and transcardially perfused with saline and 4% paraformaldehyde. After removal from the skull, brains were post-fixed in 4% paraformaldehyde for 48 h. The brains were embedded in paraffin, and coronal sections were prepared for immunostaining. The sections were washed in PBS several times and placed in 3% H_2_O_2_ for 15 min, and then incubated in 10% normal goat serum (Sigma) for 1 h to block nonspecific binding of IgG and incubated overnight at 4°C with NeuN (1:200, Millipore) and light chain 3 (LC3; 1:200, Santa Cruz) antibody. Then, the sections were washed in PBS and incubated with appropriate secondary antibody. Finally, the stained cells were observed with a fluorescent microscopy and captured using a CCD camera.

### Primary Culture of Cortical Neurons

Primary cultured cortical neurons were prepared from the cerebral cortex of day 15 C57BL/6 mouse embryos. Mice were obtained from the Experimental Animals Center, Southern Medical University. Primary cultured cortical neurons were prepared as described previously (Pavlovski et al., 2012). Briefly, cortices were dissected, mechanically minced and minced cortical tissues were then dissociated by mild trypsinization (0.02% w/v) at 37°C for 10 min followed by trituration in a DNase solution (0.004% w/v) containing a soybean trypsin inhibitor (0.05% w/v) to prepare single cell suspension. The single cell suspension was re-suspended in a DMEM medium (5 mM KCl, 31 mM glucose, and 0.2 mM glutamine) supplemented with insulin, penicillin and 10% FBS (DMEM-FBS). Then cells were plated on poly-L-lysine-coated 6-well plates at 1.0 × 10^6^ cells/ml of neurobasal medium (Gibco) with 2% B-27 (Gibco) and 0.5 mM Glutamax (Gibco) for culturing. Half of the medium was changed after the first 3 days and three times a week thereafter. Neurons were cultured for 7–9 days before experiment, which may allow percentage of neurons to reach ~90% (Hu et al., 2013).

### Identification of Primary Cultured Neurons’ Purity

β-III Tubulin, is regarded as a neuron-specific marker, which was used to verify neuronal purity by flow cytometry assay (Supplementary Figure S1). 1 × 10^6^ cells/100 μL primary cultured neurons were harvested into tubes. Cells were fixed with flow cytometry fixation buffer and permeabilized with flow cytometry permeabilization buffer. After washing, the cells were incubated with beta-III Tubulin (1:200, Proteintech) for 45 min at room temperature in the dark and subsequent analyzed by flow cytometry (BD bioscience, San Hose, CA, USA).

### Oxygen-Glucose Deprivation (OGD) and Drug Treatment

To induce OGD, the primary neurons were washed three times and incubated with glucose-free Earle’s balanced salt solution (EBSS) and placed within a hypoxic chamber (Forma Scientific, USA) which was continuously maintained with 95% N_2_ and 5% CO_2_ at 37°C to obtain 1% O_2_ (Cheon et al., 2016) for 6 h. EBSS group was exposed to EBSS in the standard incubator. OGD was terminated by replacing the medium to neurobasal medium with 2% B27 in the normoxic incubator. At the end of OGD, the treatment groups, including OGD+DEX, OGD+3-MA, OGD+DEX+3-MA, OGD+RAPA and OGD+DEX+RAPA, were administrated DEX, 3-MA, DEX combined with 3-MA and RAPA, and DEX combined with RAPA, respectively, to reach a final concentration of 1 μM DEX (Dahmani et al., 2010), 1 mM 3-MA (Shi et al., 2012) or 10 nM RAPA (Wang et al., 2012) for 4 h reoxygenation. In the groups of the Control group, Control+DEX, Control+3-MA and Control+RAPA were given saline or the same concentration of DEX, 3-MA and RAPA. In Group OGD+DEX+2ME2, a final concentration of 1 μM 2ME2 was applied given reoxygenation (Ryou et al., 2015). To evaluate the effect of DEX in the acute stage of ischemia, neurons incubated was end up at 4 h reoxygenation. All experiments were at least duplicated three times biologically.

### Cell Viability Assay

To determine the time duration of OGD and the concentration used for each drug, cell viability assay was performed (Supplementary Figure S2). The viability of the primary neurons was measured by CCK-8 assay (Dojin Laboratories, Japan). Briefly, neurons were seeded in a 96-well plate and kept in a 5% CO_2_ incubator at 37°C overnight. At the end of treatment in each group, CCK-8 assay was performed according to the manufacturer’s instruction. Absorbance was determined at 450 nm by using a microplate reader (BioTek, Winooski, VT, USA). Results are shown as percentages of the control group.

### Cell Apoptosis Assessment

The apoptosis of the neurons were stained by Annexin V/PI (Beijing 4A Biotech Co. Ltd.) according to the manufacturer’s instruction. Briefly, before analysis by flow cytometry, 5 μl of Annexin-V-Fluorescein isothiocyanate (FITC) labeling reagent and 10 μl of propidium iodide (PI) were added to the medium and the neurons were incubated at room temperature for 10 min in the dark. 1 × 10^5^ cells for each sample were analyzed a flow cytometer (BD bioscience, San Hose, CA, USA).

### Immunofluorescence

The neurons grown on coverslips were fixed by 4% methanol for 15 min and 0.25% Triton for 10 min, incubated in 2% Bovine Serum Albumin(Sigma) for 1 h to block nonspecific binding of IgG. Then the cells were reacted with primary antibodies: rabbit anti-Beclin1 (1:200, Abcam), rabbit anti-BCL-2 (1:200, Santa Cruz), mouse anti-NeuN (1:200, Millipore) at 4°C overnight followed by the appropriate secondary antibodies with a mixture of goat anti-rabbit Alexa-Fluor 488 and goat anti-mouse Alexa-Fluor 594(1:200, Invitrogen). The cells were subsequently incubated with the nuclear staining dye 4,6-diamidino-2-phenylindole (DAPI, Vector Laboratories, Burlingame, CA, Burlingame, CA, USA). Finally, the stained cells were observed with a fluorescent microscopy (Nikon ECLIPSE 80i, Japan).

### Immunoblotting

Western blot was performed as previously described (Qin et al., 2010). The brain tissues from cortex of peri-ischemic area and primary cultured cortical neurons were homogenized in lysis buffer. Ultrasonic crushed tissues for three times with low power. After reaction for 20 min on ices, the samples were centrifuged at 10,000× *g* at 4°C for 10 min and the supernatant was preserved at −80°C. Before immunoblotting, the protein concentrations were determined with a BCA detection kit (Pierce, 23225) and adjusted to the equal concentrations across different samples. Protein samples (30 μg) were separated by SDS-polyacrylamide gels, then transferred to PVDF membranes. After blocked with 5% fat free milk, the membranes were incubated with a p62 (1:750, Cell Signaling), LC3 (1:500, Santa Cruz), Beclin 1 (1:1000, Abcam), BCL-2 (1:1000, Santa Cruz) and HIF-1α (1:1000, Cell Signaling), the mammalian target of rapamycin (mTOR), p-mTOR (1:1000, Sigma) and β-actin (1:5000, Cell Signaling). Blots were visualized using an Image Lab 5.1 software (Bio-Rad, Hercules, CA, USA) and were analyzed using ImageJ.

### Statistical Analysis

Data are expressed as mean ± SEM. Differences were evaluated by one-way analysis of variance (ANOVA; three or more groups). When only two groups were compared, an unpaired *t*-test was used. *P* < 0.05 was considered statistical significance. Statistical analyses were performed using SPSS 20.0 Statistics (IBM SPSS Statistics for Version 20.0, IBM Corp, North Castle, NY, USA).

## Results

### DEX Improved Infarction and Reduced Neurological Scores after tMCAO

**Figure 1:**
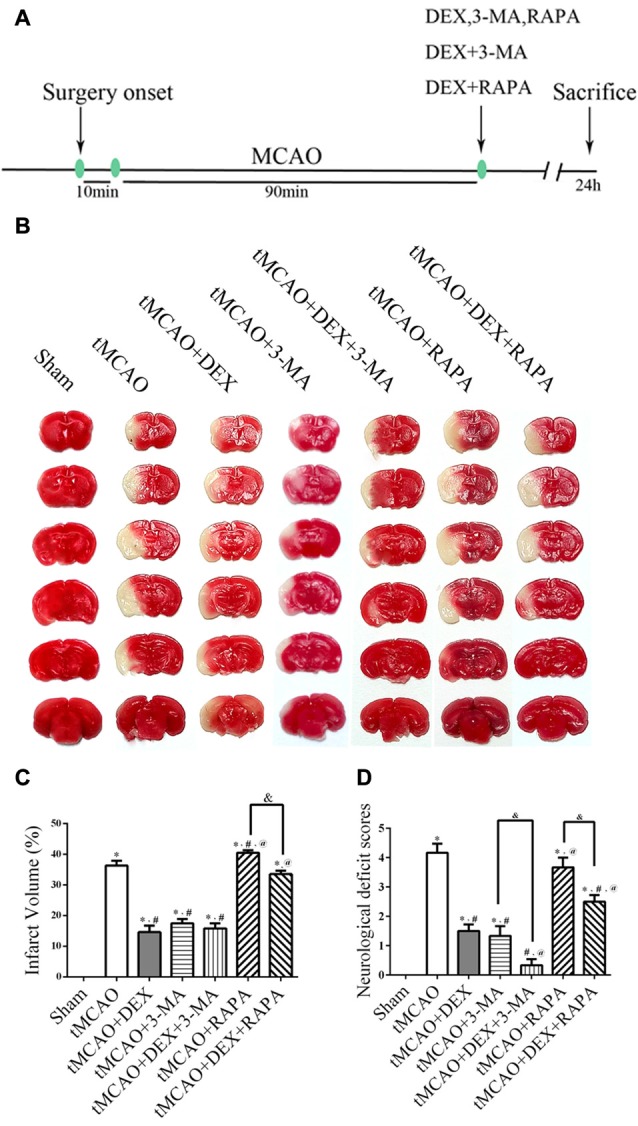
Dexmedetomidine (DEX) improved infarction and reduced neurological scores after transient middle cerebral artery occlusion (tMCAO). **(A)** Schematic of *in vivo* experiments on male C57/BL6 mice. DEX, 3-methyladenine (3-MA), RAPA, the combination of DEX and 3-MA, and the combination of DEX and RAPA were injected at the onset of reperfusion.** (B)** Representative 2,3,5-Triphenyltetrazolium chloride (TTC)-stained brain slices from each group are shown. **(C)** DEX or autophagy flux inhibitors 3-MA significantly decreased the infarct volume and post-treatment with the autophagy-inducer RAPA had no protective effect. **(D)** DEX improved neurological scores after tMCAO and this protection could be enhanced or attenuated by 3-MA or RAPA, respectively. ^*,#,@^*P* < 0.05, compared with Sham, tMCAO, tMCAO+DEX respectively; ^&^*P* < 0.05, compared between two indicated groups.

The experimental plan was showed in Figure 1A. We first examined whether DEX reduce infarction and improve neurological scores at 1 day after tMCAO. Furthermore, we utilized 3-MA and RAPA to investigate whether neuroprotection of DEX after tMCAO was related with regulation of autophagy. The data showed that treatment of DEX or autophagy flux inhibitor, 3-MA, significantly reduced the brain infarct volume. Moreover, post-treatment with the autophagy inducer, RAPA, was not protective and DEX could significantly reversed the brain infarction even when combined with RAPA (Figures 1B,C). Similarly, neurological deficit scores showed that DEX improved neurological outcomes after tMCAO, and this protection could be enhanced or attenuated by 3-MA or RAPA, respectively (Figure 1D). These data showed that DEX may regulate autophagy to produce neuroprotection in ischemic brain injury.

### DEX Inhibited Neuronal Autophagy in Mouse Cortex after tMCAO

**Figure 2:**
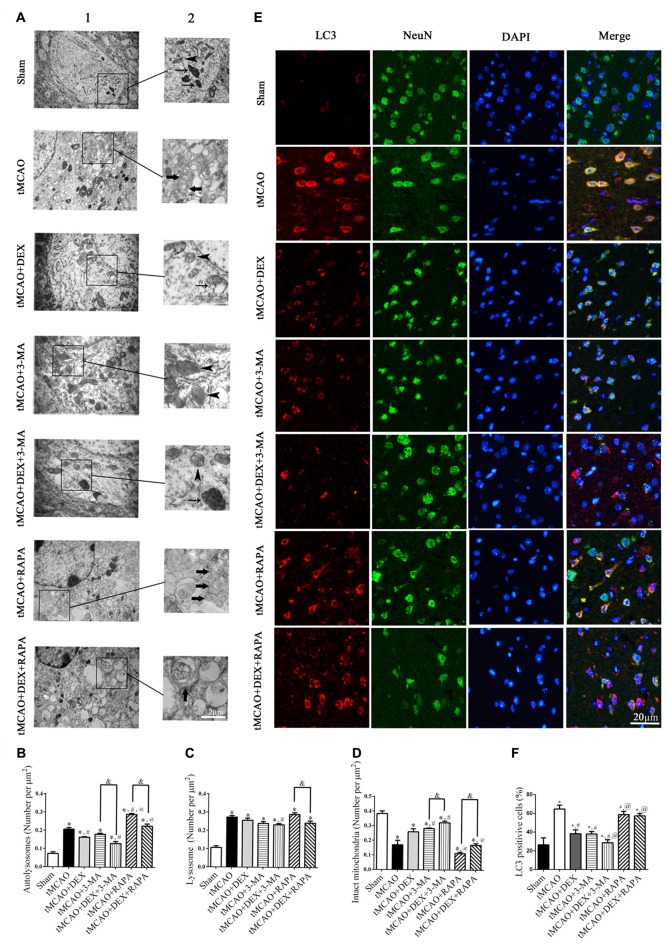
DEX inhibited neuronal autophagy in mouse cortex after tMCAO. **(A)** Ultrastructural changes in mouse cortical neurons were examined with transmission electron microscopy at 1 day after tMCAO (A1). Higher magnification of ultrastructural changes was shown in the black box for all groups (A2). Broad arrows represent the double membrane-surrounded autophagosomes; Narrow arrows represent lysosomes; Arrow heads represent mitochondria. **(B–D)** The DEX-induced neuroprotection was correlated with attenuation of cortex neuronal autophagy and the number of autophagosomes was reduced or increased when treated with 3-MA or RAPA, respectively.** (E)** Light chain 3 (LC3; red) was present in NeuN-positive cells (green) and cell nuclei were counterstained by 4,6-diamidino-2-phenylindole (DAPI; blue) in mouse cortex after tMCAO.** (F)** The immunofluorescence intensity of LC3 in NeuN was reduced after tMCAO and decreased or increased when combined with 3-MA or RAPA, respectively. ^*,#,@^*P* < 0.05, compared with Sham, tMCAO, tMCAO+DEX respectively; ^&^*P* < 0.05, compared between two indicated groups.

To clearly evaluate the effect of DEX, 3-MA and RAPA on autophagy, ultrastructural changes in mouse cortical neurons were examined with transmission electron microscopy at 1 day after tMCAO (Figure 2A). The number of intact mitochondria (arrow heads), autophagosomes (broad arrows) and lysosomes (narrow arrows) in each group of cortical neurons were counted and analyzed (Figures 2B–D). In neurons, DEX, 3-MA or their combination treatment after tMCAO respectively increased intact mitochondria and decreased autophagosomes dramatically compared with tMCAO group, but RAPA treatment after tMCAO decreased intact mitochondria and increased autophagosomes significantly and this effect could be partly reversed by DEX. To confirm whether DEX inhibited neuronal autophagy to produce neuroprotection after tMCAO, we elucidated the distribution pattern of LC3 positive cells in neurons by tissue immunostaining. Our results revealed that DEX, 3-MA and DEX+3-MA significantly decreased LC3 positive cells compared with tMCAO group. Meanwhile, RAPA and the combination of RAPA and DEX after tMCAO respectively had no effect on the distribution pattern of LC3 positive cells compared with tMCAO group (Figures 2E,F). Consequently, our results indicated that DEX inhibited neuronal autophagy to produce neuroprotection at 1 day after tMCAO.

### DEX Increased Viability and Inhibited Apoptosis of Neurons Following OGD

**Figure 3:**
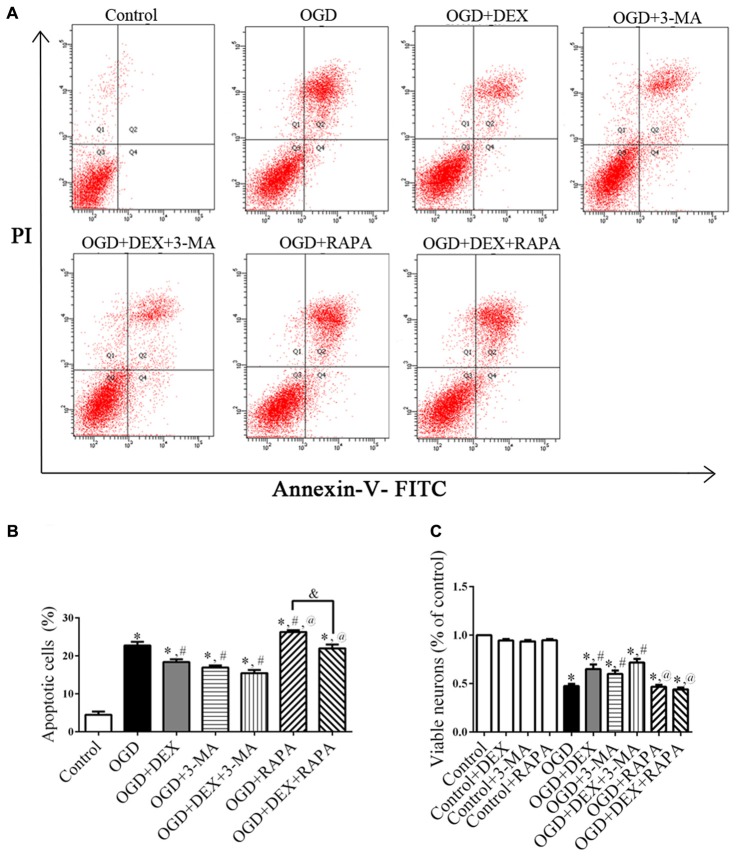
DEX increased viability and inhibited apoptosis of neurons following oxygen-glucose deprivation (OGD). **(A)** The effects of DEX, 3-MA and RAPA on neurons apoptosis after OGD were measured by flow cytometry.** (B)** DEX inhibited apoptosis of neurons following OGD and this protection could be enhanced or attenuated by 3-MA or RAPA, respectively. **(C)** DEX or autophagy flux inhibitors 3-MA increased the viability of neurons following OGD. Moreover, the autophagy-inducer RAPA produced almost none protective effect. ^*,#,@^*P* < 0.05, compared with Control, OGD, OGD+DEX respectively; ^&^*P* < 0.05, compared between two indicated groups.

Because autophagy and apoptosis could occur in the same injured neurons (Tian et al., 2010), we next investigated whether DEX affected cell apoptosis or viability using flow cytometry and CCK-8 assay. Flow cytometry detection revealed that DEX, 3-MA and DEX+3-MA significantly inhibited apoptosis of neurons following OGD compared with OGD group (Figures 3A,B). Then, CCK-8 assay results revealed that DEX, 3-MA and DEX+3-MA significantly increased viability. Meanwhile, RAPA and the combination of RAPA and DEX had no effect on viability of neurons following OGD (Figure 3C). Collectively, DEX increased the viability and inhibited the apoptosis of neurons following OGD, and this protection could be enhanced or attenuated by 3-MA or RAPA, respectively.

### DEX Attenuated the Activation of Autophagy in Neurons Following OGD

**Figure 4:**
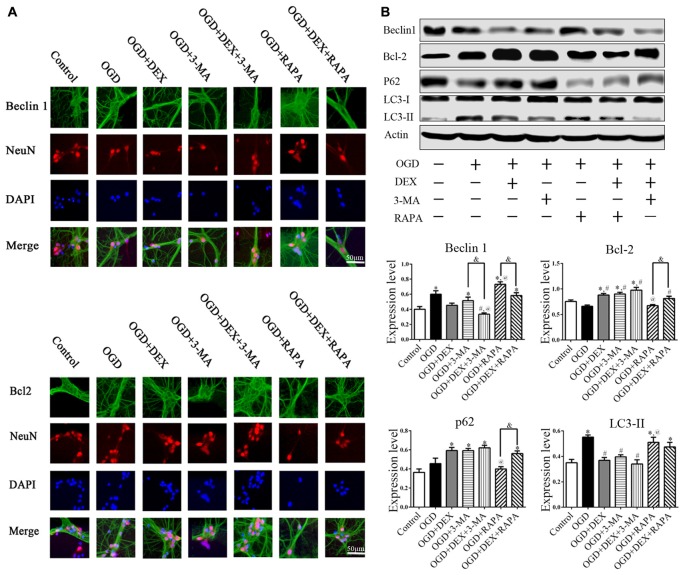
DEX attenuated autophagy of primary cultured cortical neurons following OGD. **(A)** Beclin1, Bcl-2 immunoreactivity (green) was present in NeuN-positive cells (red) and cell nuclei were counterstained by DAPI (blue). **(B)** Cell immunoblotting results showed DEX attenuated the activation of autophagy in neurons following OGD. The autophagic inhibition effect of DEX was enhanced or attenuated by 3-MA or RAPA, respectively. ^*,#,@^*P* < 0.05, compared with Control, OGD, OGD+DEX respectively; ^&^*P* < 0.05, compared between two indicated groups.

To examine the autophagy in primary neurons, we confirmed the expression of autophagy related proteins by cell immunofluorescence and immunoblotting. These data showed that DEX, 3-MA and DEX+3-MA treatment significantly increased Bcl-2 and decreased Beclin 1 expression respectively in cortical neurons following OGD compared with OGD group. RAPA had no effect on Bcl-2 and Beclin 1 expression level, but the Bcl-2 increased and Beclin 1 decreased, respectively when combined with DEX (Figure 4). Additionally, the expression of other autophagic proteins, p62 and LC3, were increased and decreased respectively by DEX in cortical neurons following OGD with or without the combination with 3-MA or RAPA (Figures 4A,B). These results indicate DEX attenuated the activation of autophagy in neurons following OGD. The autophagic inhibition effect of DEX was enhanced or attenuated by 3-MA or RAPA, respectively.

### DEX Inhibited Autophagy Following OGD and tMCAO through Upregulation of HIF-1α

**Figure 5:**
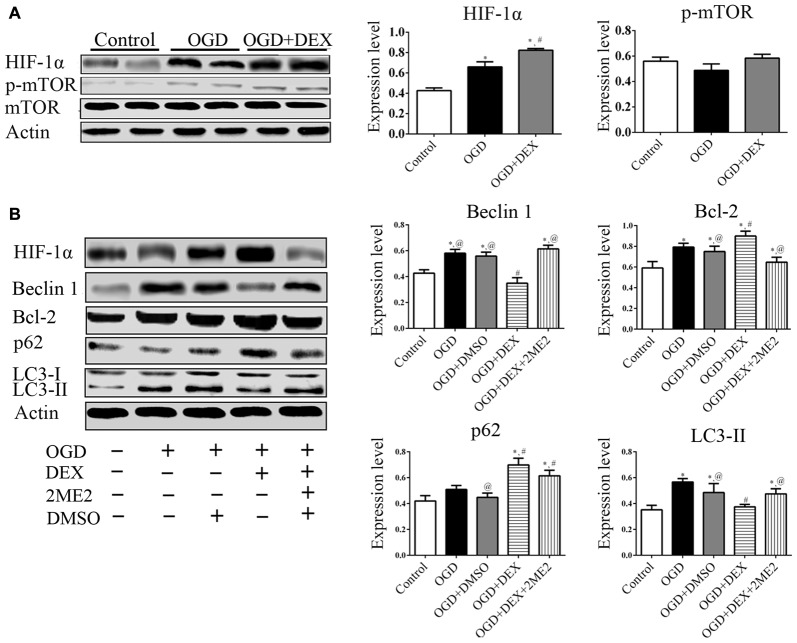
DEX inhibited autophagy following OGD related with upregulation of hypoxia-inducible factor-1α (HIF-1α) in neurons. **(A)** HIF-1α was significantly up-regulated after DEX treatment following OGD. **(B)** The immunoblotting results suggested the autophagy inhibition of DEX was reversed by 2-Methoxyestradiol (2ME2) following OGD. ^*,#,@^*P* < 0.05, compared with Control, OGD, OGD+DEX, respectively.

**Figure 6:**
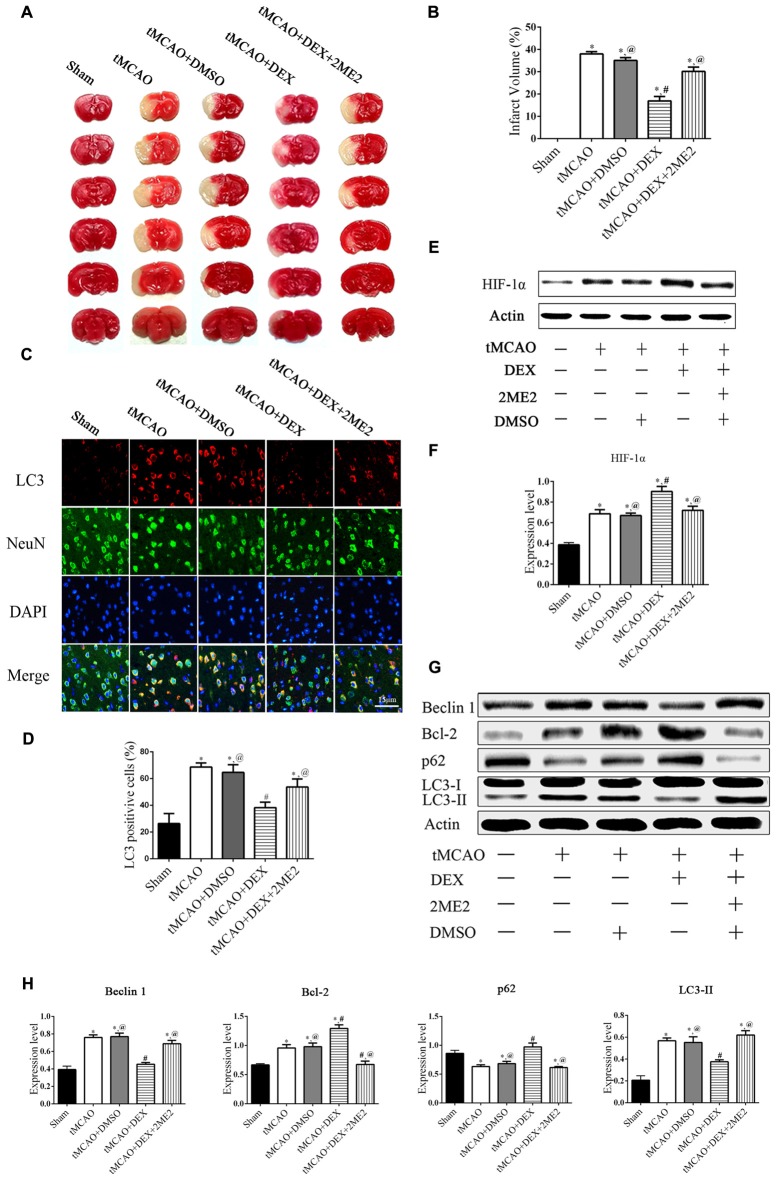
DEX produced neuroprotection and autophagic inhibition of mouse cortex after tMCAO via HIF-1α. **(A)** The effect of treated with DEX+2ME2 followed by ischemia reperfusion injury on infarct size. **(B)** The neuroprotection of DEX could be reversed by 2ME2 after tMCAO. **(C)** LC3 (red) was present in NeuN-positive cells (green) and cell nuclei were counterstained by DAPI (blue) of mouse cortex after tMCAO. **(D)** LC3 immunofluorescence intensity in NeuN was higher after DEX combination of 2ME2 than DEX alone. **(E,F)** HIF-1α was significantly up-regulated after DEX treatment following tMCAO. **(G)** Western blots of the effects of treated with DEX+2ME2 followed by ischemia reperfusion injury on autophagic proteins after tMCAO. **(H)** The immunoblotting results suggested the autophagy inhibition of DEX were reversed by 2ME2 after tMCAO. ^*,#,@^*P* < 0.05, compared with Sham, tMCAO, tMCAO+DEX, respectively.

To examine which signaling pathways involved in the DEX-mediated autophagy, we measured expression of mTOR and HIF-1α following OGD. In the neurons, the expression of HIF-1α in OGD group and OGD+DEX group was elevated compared with control group, with an enhanced elevation in DEX+OGD group than in OGD group. DEX had no effect on p-mTOR/mTOR expression in neurons following OGD (Figure 5A). Furthermore, 2ME2, an inhibitor of HIF-1α, was applied to determine whether DEX inhibit autophagy was caused by up-regulation of HIF-1α. The immunoblotting results suggested that the DEX-induced inhibition of autophagy was reversed by 2ME2 (Figure 5B). To confirm that the neuroprotection effect of DEX could be neutralized by 2ME2, we tested the effects of 2ME2 following stroke and DEX treatment in the ischemic brain. Similar to the results *in vitro*, infarct volume showed an enlargement in DEX+2ME2 group than DEX group (Figures 6A,B). Moreover, our immunostaining results from brain section demonstrated that the number of LC3 positive cells decreased in the DEX after ischemia-reperfusion injury. When using 2ME2 to block the HIF-1α, the number of LC3 positive cells increased compared to DEX group (Figures 6C,D). The immunoblotting results from brain tissues also suggested that HIF-1α was significantly up-regulated after DEX treatment following tMCAO (Figures 6E,F). DEX-induced inhibition of autophagy were reversed by 2ME2 after tMCAO (Figures 6G,H). Collectively, our *in vitro* and *in vivo* results suggested up-regulation of HIF-1α may play a key role in DEX-mediated inhibition of autophagy.

## Discussion

**Figure 7:**
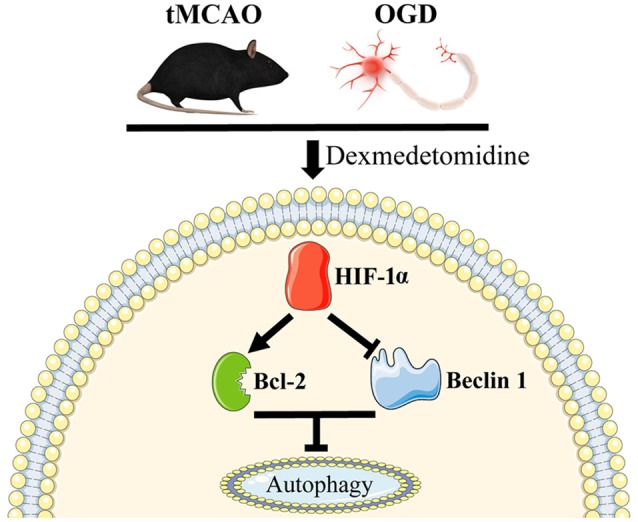
Schematic mechanism of DEX in regulation of autophagy. DEX inhibits neuronal autophagy via up-regulating HIF-1α.

In this study, we presented that DEX-induced inhibition of neuronal autophagy was neuroprotective at 1 day after tMCAO and this protection could be enhanced or attenuated by 3-MA or RAPA, respectively. *In vitro* studies confirmed that DEX treatment or DEX combined with 3-MA or RAPA showing the same effect on regulating neuronal autophagy. Furthermore, our results found that HIF-1α was up-regulated *in vivo* and *in vitro*. When HIF-1α up-regulation was inhibited by 2ME2, the DEX-induced inhibition of autophagy was ameliorated. Thus, our *in vitro* and *in vivo* results demonstrated that DEX inhibited neuronal autophagy to produce neuroprotection effect after cerebral ischemic-reperfusion injury, and this neuroprotection effect was related with DEX-induced HIF-1α expression (Figure 7).

Ischemia-mediated brain lesions occur and develop rapidly (Yenari and Han, 2012). The interruption of CBF produces a quick neuronal stress in response to hypoxia (Fedorovich et al., 2017). Even if the blood supply is restored, the damage by reperfusion is not negligible, and more neurons will be damaged. Gan et al. (2014) found that anti-inflammatory intervention at 6 h after tMCAO led to obviously smaller infarct size and less neurological deficits than those in their counterparts given the same kind of treatment within the first 12 h after stroke. Although several studies have revealed that DEX has neuroprotective and anti-inflammatory properties at the beginning of the stroke, the role of autophagy remains unclear (Yenari and Han, 2012; Kim et al., 2017). Autophagy is a complex mechanism to degrade cellular components process. Initially, autophagy has been considered as a vital physiological mechanism to maintain cellular homeostasis. Tian’s group adopted a novel *in vivo* technique depicted the detailed autophagic process after cerebral ischemia (Tian et al., 2010) Their results found that tMCAO activated autophagy both in neurons and glia resident in ischemic core and penumbra area. They also pointed out the autophagy peaked at 1 day after tMCAO. Moreover, the number of autophagic neurons is almost two times of the autophagic astrocytes and most of the autophagic neurons are in penumbra area while the co-existed apoptosis and autophagy in the same neurons implied a relationship between autophagy and apoptosis. Thus, in current study we focused on the neuronal autophagic status in the peri-ischemic area at 1 day after tMCAO.

Our results showed tMCAO activated neuronal autophagy at 1 day after tMCAO while the neuroprotection of post-conditioning with DEX correlated with attenuation of cortex neuronal autophagy. Meanwhile, post-conditioning with autophagy inhibitor (3-MA) alone or combined with DEX also confirmed that inhibition of neuronal autophagy was neuroprotective. Although our results are consistent with previous report (Puyal et al., 2009; Wang et al., 2011), other researchers presented different conclusion with us. Carloni et al. (2010) found pre-conditioning neonatal rats with hypoxia-ischemia to induce autophagy could produce comparable neuroprotection when pre-conditioning with RAPA. Qin’s group also suggested that activation autophagy by cerebral ischemic pre-conditioning can produce equivalent neuroprotection to autophagic inducer, RAPA (Qin et al., 2010). However, these discrepancies may result from the different conditioning strategy (pre- or post-) and different ischemic model (permanent or transient) adopted in their study and ours. Additionally, Wang et al. (2012) found that 3-MA could partly abolish the LC3 II expression and remarkably increase the damage of neurons, whereas RAPA increased LC3 II expression and improved neuronal viability at 2 h after only 2 h exposed to OGD. In addition, the time of reperfusion injury and the time of primary neurons exposed to OGD are also different from our experiments. More importantly, autophagy and apoptosis could occur in the same injured neurons (Tian et al., 2010), and the status of autophagy may increase or decrease neuronal apoptosis at different timepoints and conditions (Tolkovsky et al., 2002; Ginet et al., 2009). Therefore, autophagy may act differently in cells survival when exposed to deleterious stimuli (Puyal et al., 2009; Wang et al., 2012) and the detailed effect and mechanism of autophagic equilibrium in cell homeostasis, especially “when”, “where” and “how”, during cerebral ischemia still need further investigation.

Autophagy is a multi-step dynamic process that should be monitored by characteristic proteins at different stages. LC3 is shifted from a LC3-I to a LC3-II, which indicates the beginning of autophagy and formation of autophagosomes (Papadakis et al., 2013). p62/SQSTM1 binds directly to LC3 and is degraded by the autophagic-lysosomal pathway, implying a complete autophagic flux (Komatsu et al., 2007). Our results showed OGD increased neuronal LC3-II expression while down-regulated p62 expression. We also found that post-conditioned with DEX in OGD neurons significantly decreased LC3-II expression level while increased p62 accumulation compared with OGD group. In addition, autophagy gene *beclin1*, a mammalian homolog of Atg6, interact with the antiapoptotic protein Bcl-2, implying autophagy and apoptosis could occur in the same injured cells (Pattingre et al., 2005). Similarly, we detected Bcl-2 and Beclin1 expression after OGD injury in neurons, and found DEX led to a significantly up-regulated expression of Bcl-2 while down-regulated expression of Beclin1 compared with OGD group. Taken together, our data demonstrated that DEX inhibited neuronal autophagy in mice cortex after OGD model of primary cultured cortical neurons.

Following OGD injury, mTOR expression is the main factor contributing to the progression of neuronal cell autophagy and the subsequent cell survival (Hu et al., 2015). However, our results indicated that DEX had no effect on p-mTOR/mTOR expression in neurons following OGD, indicating DEX-induced neuroprotection effect is mTOR-independent. Besides mTOR, HIF-1α plays a crucial role by upregulating its targets expression, BNIP3 and BNIP3L, to activate autophagy in tumor cells (Bellot et al., 2009; Mazure and Pouyssegur, 2010). In addition, hypoxia promotes PDGFR-induced autophagy in tumor cells dependent on HIF-1α signaling while independent of BNIP3 and BNIP3L (Wilkinson et al., 2009). Emerling’s group also suggested that HIF-1α can activate AMPK and regulate autophagy (Emerling et al., 2009). In fact, these studies have reported HIF-1α promoted autophagy via different pathways whereas we, for the completely different cell types, demonstrate that HIF-1α promotes neurons cell survival by inhibiting neuronal autophagy when post-conditioning with DEX treatment. Blocking the HIF-1α signaling pathway with 2ME2 neutralized the neuroprotection by DEX-induced autophagy inhibition. Together, our results showed that HIF-1α in neurons may play a critical role in DEX-mediated inhibition of autophagy.

In conclusion, for the first time, we showed that DEX mediates autophagy of cortical neurons to produce neuroprotection after OGD and tMCAO models via upregulation of HIF-1α. Our findings indicated that DEX treatment at the onset of reperfusion may have important clinical relevance, revealing new mechanisms of DEX in the stroke brain. Nevertheless, more investigations are needed to elucidate the effect and detailed signaling pathways involved in DEX-produced inhibition of autophagy at different timepoints. Furthermore, the effects of DEX on autophagy status of glia, e.g., astrocytes, after cerebral ischemia and the potential mechanism is still unclarified.

## Author Contributions

CL and M-WO carried out most procedures of this study, participated in the data analysis and manuscript writing. Y-YF and S-JL participated in design of animal study and interpreted part data of this study. QZ and JF participated in the collection of data, data analysis. TT and Z-SQ designed this study, provided financial support and wrote the manuscript. All authors read and approved the final manuscript.

## Conflict of Interest Statement

The authors declare that the research was conducted in the absence of any commercial or financial relationships that could be construed as a potential conflict of interest.

## Supplementary Material

FIGURE S1 Identification of primary cultured neurons’ purity. Neurons were cultured for 7–9 days and then stained with mouse anti-β-III tubulin APC-conjugated monoclonal antibody. **(A)** Single suspension of primary cultured neurons was first gated for forward scatter (FSC), a measure of size, and side scatter (SSC), a measure of cell granularity. **(B)** Flow cytometry assay revealed that the majority of these cells (>90%) were β-III Tubulin positive from 80% gated intact cells.

FIGURE S2 Effects of oxygen-glucose deprivation (OGD) and dexmedetomidine (DEX) post-conditioning on the cell viability. **(A)** Six hours was chosen as the OGD period applied in all experiments. **(B)** The neuroprotective effect of DEX was maximum for the 4 h reperfusion period after the end of OGD. **P* < 0.05, compared with Control; ^&^*P* < 0.05, compared between two indicated groups.
