# Supplementary figures and images for: Dexmedetomidine Protects Mouse Brain from Ischemia-Reperfusion Injury via Inhibiting Neuronal Autophagy through Up-Regulating HIF-1α
Source: Front Cell Neurosci. 2017 Jul 6;11:197. doi: 10.3389/fncel.2017.00197 (PMC5498477; doi:10.3389/fncel.2017.00197)

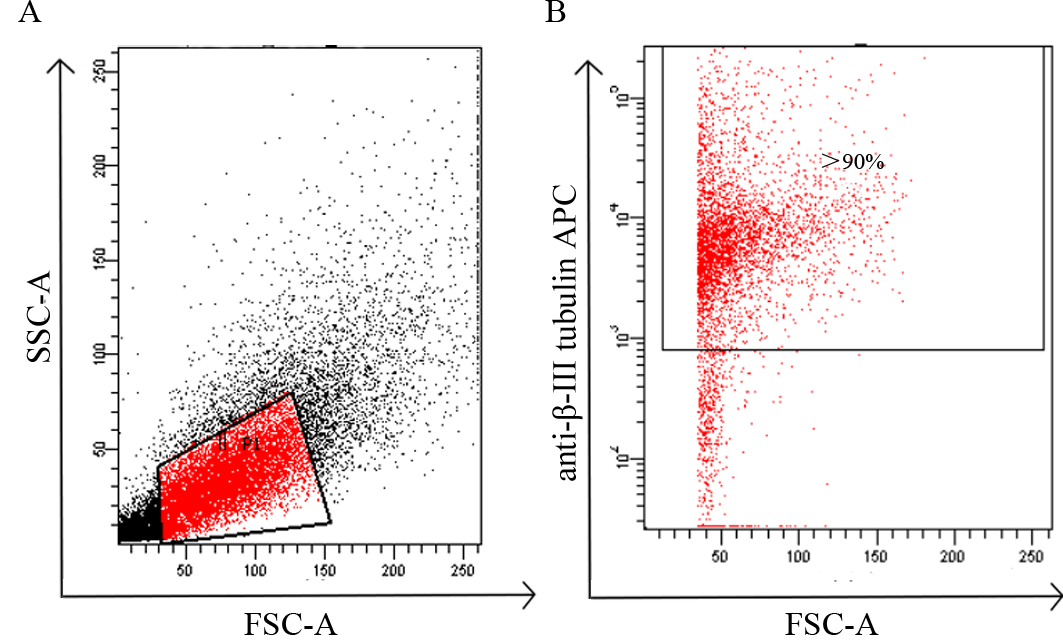

Supplement: FIGURE S1 — Identification of primary cultured neurons’ purity. Neurons were cultured for 7–9 days and then stained with mouse anti-β-III tubulin APC-conjugated monoclonal antibody. (A) Single suspension of primary cultured neurons was first gated for forward scatter (FSC), a measure of size, and side scatter (SSC), a measure of cell granularity. (B) Flow cytometry assay revealed that the majority of these cells (>90%) were β-III Tubulin positive from 80% gated intact cells. [file Image_1.tif]

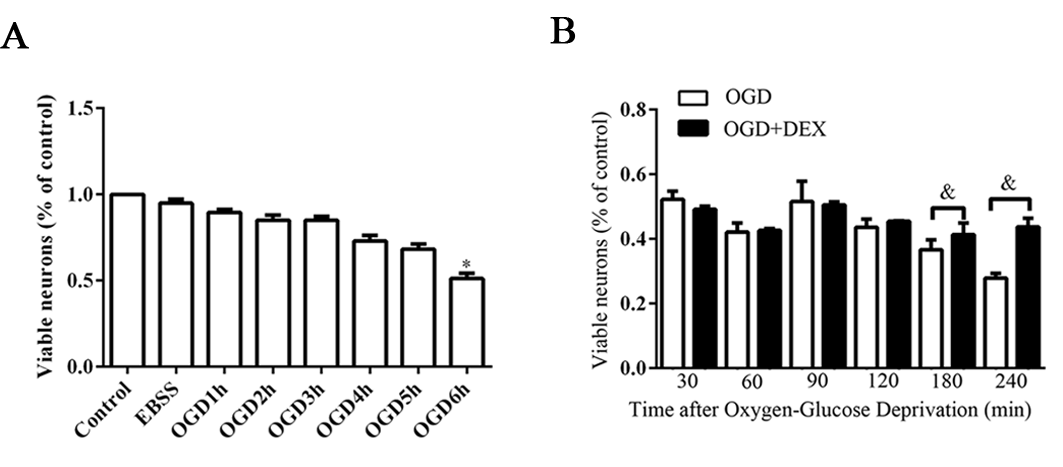

Supplement: FIGURE S2 — Effects of oxygen-glucose deprivation (OGD) and dexmedetomidine (DEX) post-conditioning on the cell viability. (A) Six hours was chosen as the OGD period applied in all experiments. (B) The neuroprotective effect of DEX was maximum for the 4 h reperfusion period after the end of OGD. *P < 0.05, compared with Control; &P < 0.05, compared between two indicated groups. [file Image_2.tif]
